# Effect of Heat Processing on IgE Reactivity and Cross‐Reactivity of Tropomyosin and Other Allergens of Asia‐Pacific Mollusc Species: Identification of Novel Sydney Rock Oyster Tropomyosin Sac g 1

**DOI:** 10.1002/mnfr.201800148

**Published:** 2018-06-21

**Authors:** Jennifer M. Rolland, Nirupama P. Varese, Jodie B. Abramovitch, Jessica Anania, Roni Nugraha, Sandip Kamath, Anita Hazard, Andreas L. Lopata, Robyn E. O'Hehir

**Affiliations:** ^1^ Department of Immunology and Pathology Monash University Melbourne Victoria Australia; ^2^ Department of Allergy Clinical Immunology and Respiratory Medicine Central Clinical School Monash University Melbourne Victoria Australia; ^3^ Centre for Biodiscovery and Molecular Development of Therapeutics Molecular Allergy Research Laboratory James Cook University Townsville Australia; ^4^ Department of Aquatic Product Technology Bogor Agricultural University Bogor Indonesia

**Keywords:** heat processing, IgE cross‐reactivity, mollusc allergen, Sydney rock oyster, tropomyosin

## Abstract

**Scope:**

Shellfish allergy is an increasing global health priority, frequently affecting adults. Molluscs are an important shellfish group causing food allergy but knowledge of their allergens and cross‐reactivity is limited. Optimal diagnosis of mollusc allergy enabling accurate advice on food avoidance is difficult. Allergens of four frequently ingested Asia‐Pacific molluscs are characterized: Sydney rock oyster (*Saccostrea glomerata*), blue mussel (*Mytilus edulis*), saucer scallop (*Amusium balloti*), and southern calamari (*Sepioteuthis australis*), examining cross‐reactivity between species and with blue swimmer crab tropomyosin, Por p 1.

**Methods and results:**

IgE ELISA showed that cooking increased IgE reactivity of mollusc extracts and basophil activation confirmed biologically relevant IgE reactivity. Immunoblotting demonstrated strong IgE reactivity of several proteins including one corresponding to heat‐stable tropomyosin in all species (37–40 kDa). IgE‐reactive Sydney rock oyster proteins were identified by mass spectrometry, and the novel major oyster tropomyosin allergen was cloned, sequenced, and designated Sac g 1 by the IUIS. Oyster extracts showed highest IgE cross‐reactivity with other molluscs, while mussel cross‐reactivity was weakest. Inhibition immunoblotting demonstrated high cross‐reactivity between tropomyosins of mollusc and crustacean species.

**Conclusion:**

These findings inform novel approaches for reliable diagnosis and improved management of mollusc allergy.

## 1 Introduction

Shellfish allergy is an increasingly important global health priority, often presenting in childhood and typically persisting into adulthood.^1^, ^2^, ^3^ Reactions range from mild irritation to life‐threatening anaphylaxis,^4^ currently with only emergency treatment and no allergen‐specific therapy available.^5^, ^6^ Molluscs are a major group of shellfish causing food allergy, but in many studies they are combined with crustaceans under the term shellfish.^7^ Consequently, mollusc allergy is clinically under‐reported and allergens are ill‐defined. To date only four mollusc allergens are listed in the WHO/International Union of Immunological Societies (IUIS) Allergen Nomenclature Sub‐Committee database, all of which are different tropomyosins (http://www.allergen.org/index.php). Additional mollusc allergens have been reported, but not yet fully characterized.^8^, ^9^ Elucidation of the full range and immunoreactivity of mollusc allergens will facilitate more precise diagnostic tools and much needed specific immunotherapy.

As for other shellfish groups, tropomyosin has been identified as a major allergen for several mollusc species.^10^, ^11^, ^12^, ^13^, ^14^, ^15^ It is a highly water‐soluble and heat‐stable protein, found in both muscle and non‐muscle cells. Other allergens identified in mollusc species include paramyosin, hemocyanin, myosin heavy chain, amylase, arginine kinase, and triosephosphate isomerase.^8^, ^9^ However, the clinical importance and cross‐reactivity of these allergens are unclear. Patients frequently show clinical reactivity to both mollusc and crustacean shellfish species. It has not been established whether this is true IgE cross‐reactivity or simply co‐sensitization to mollusc and crustacean allergens. While crustacean tropomyosins show very high amino acid sequence identity (up to 98%) with IgE cross‐reactivity as demonstrated by us and others,^16^, ^17^ the reported sequence identity between crustacean and mollusc tropomyosins is only 56–68%.^13^ Whether there is sufficient homology at IgE binding epitopes for clinically relevant cross‐reactivity is unknown.

Food processing, including heating and digestion, can affect IgE reactivity of allergens by chemically altering protein structures via different processes including aggregation, polymerization, and degradation.^18^, ^19^, ^20^ Heating can also alter conformation of proteins and hence allergenicity by disrupting disulfide bonds. Tropomyosin has no disulfide bonds, consistent with its relative heat stability,^21^, ^22^ but the impact of heating on other mollusc allergens is ill‐defined. Nakamura and colleagues showed that particular sugars influenced the effect of the Maillard reaction during heating on the allergenicity of tropomyosin from two mollusc species with conflicting results: allergenicity of squid tropomyosin was decreased and scallop tropomyosin increased.^23^, ^24^

We report here the characterization of allergens in four commonly consumed Asia‐Pacific mollusc species: *Saccostrea glomerata* (Sydney rock oyster, SRO), *Mytilus edulis* (blue mussel), *Amusium balloti* (saucer scallop), and *Sepioteuthis australis* (southern calamari). SRO allergens were identified by mass spectrometry, the novel oyster tropomyosin Sac g 1 was sequenced, and cross‐reactivity with other mollusc and crustacean tropomyosins assessed. The effect of heat treatment of mollusc extracts on IgE reactivity was also examined. The findings of this study will inform novel and reliable diagnostic approaches to enhance management of mollusc allergy.

## 2 Experimental Section

### 2.1 Study Population, Sera, and Ethics Statement

Sera were obtained from 13 patients recruited from the Alfred Hospital Allergy Clinic with a clinical history of seafood allergy and positive mollusc‐specific IgE by ImmunoCAP (>0.35 kUA L^–1^; Phadia Pty Ltd, Uppsala, Sweden; **Table** 1; mean age 33.8 ± 9.2 years; 10 F/3 M). Two nonatopic control subjects were recruited with negative skin prick tests to a panel of common aeroallergens and no clinical history of seafood allergy. A non‐shellfish allergic, atopic control subject with negative seafood‐ and house dust mite–specific IgE, and positive ryegrass pollen–specific IgE was included. The study was approved by the Alfred Hospital Research Ethics Committee (Project number 192/07) and the Monash University Human Ethics Committee (MUHREC CF08/0225) and informed written consent was obtained from each subject.

**Table 1:** Clinical characteristics of mollusc‐sensitized, seafood‐allergic subjects

No.	Sex	Age	Total IgE [IU mL^–1^]	Specific IgE [kU_A_ L^–1^]				Clinical presentation	
				Oyster	Mussel	Scallop	Calamari	Symptoms with	Symptoms
A1	F	20	242	0.42	0.10	0.18	0.55	Prawn	As, R, U
A2	F	34	199	0.93	0.57	1.93	4.19	Prawn, crab, calamari, lobster	R, U, A, An
A3	F	31	2231	0.09	0.05	0.53	0.22	Prawn, crab, fish	As, R, An, U
A4	F	26	1946	0.92	0.61	1.13	1.07	Flounder, prawn, crab	R, A, An
A5	M	45	976	2.04	9.27	5.10	10.2	Calamari, snapper, tuna	R, An
A6	F	33	28	3.75	2.84	3.00	5.21	Shellfish, scallops, oyster	U, An, pO
A7	M	44	140	4.29	0.07	0.17	0.02	Flake, sea perch, rockling	pO, An
A8	M	33	183	1.04	0.98	0.46	1.36	Mussel, scallops	An
A9	F	45	167	5.99	NT	NT	NT	Shellfish	As, R, U, An
A10	F	32	130	2.41	NT	NT	NT	Crab	An
A11	F	48	579	1.11	1.09	1.99	2.63	Crustaceans/molluscs	R, H, A
A12	F	24	266	6.68	NT	NT	NT	Salmon, crab, lobster, shrimp	An
A13	F	24	227	2.59	0.63	4.66	1.95	Prawns, calamari, fish	An

NT, not tested; As, asthma; R, rhinitis; U, urticaria; A, anaphylaxis; An, angioedema; pO, periorbital edema; H, hypotension.

### 2.2 Mollusc Extract Preparation

Flesh from fresh Sydney rock oyster (*S. glomerata*) with (raw oyster, RO) or without visceral mass (ROvm), blue mussel (*M. edulis*; RM), saucer scallop (*A. balloti*; RS), and southern calamari (*S. australis*; RC) was removed from its shell, sliced into fine pieces and incubated in PBS pH 7.2 overnight at 4 °C with constant mixing. After centrifugation at 13 000 × *g* at 4 °C for 20 min, the supernatant was collected, filter‐sterilized, and stored at −80 °C. For cooked SRO with (CO) or without visceral mass (COvm), blue mussel (CM), saucer scallop (CS), and southern calamari (CC) extracts, the whole molluscs were cooked in boiling PBS for 20 min prior to extract preparation as above. Extract protein concentrations were determined using the Bradford assay kit (Bio‐Rad Laboratories, Hercules, CA).

### 2.3 IgE ELISA and Inhibition ELISA

IgE ELISA was performed as described previously.^16^ Briefly, 1 μg mL^−1^ extract was coated onto 96‐well EIA/RIA plates (Costar, St. Louis, MO) and screened with patient sera diluted 1:10 in 1% skim milk powder/0.05% Tween/PBS. IgE binding was detected using rabbit anti‐human IgE (1:4000 dilution; Dako, Glostrup, Denmark) and goat anti‐rabbit IgG‐HRP (1:1000 dilution; Promega, Madison, WI).

Inhibition IgE ELISA was performed as described^16^ using sera previously found positive (O.D._450 nm_ ≥ 1.5 at 1:10 dilution) by direct ELISA for the relevant coating extract: for CC—A8, A10, A13; CM—A8, A11; CS—A8, A10, A13; CO—A7, A8, A10, A11, A13, and COvm—A2, A8, A10, A13. Sera were first titrated for IgE reactivity with the mollusc extract to determine the serum concentration at which the O.D._450 nm_ was ≈1 and within the linear phase of the titration curve. Using this dilution, patient sera were preincubated with increasing concentrations of mollusc extracts or ovalbumin (OVA, negative control) for 1 h at room temperature before testing by ELISA as above. The percentage inhibition was calculated as 100 − [(O.D._450 nm_ of serum with inhibitor/O.D._450 nm_ of serum without inhibitor) × 100]. To allow comparison between patients, the concentration required for 50% inhibition of IgE binding was determined. To assess nonspecific inhibition by mollusc extracts, serum from a non‐seafood allergic, atopic (ryegrass pollen‐sensitized) subject was incubated with the above inhibitors, and then tested for IgE reactivity with ryegrass pollen extract in comparison with untreated serum.

### 2.4 Basophil Activation Test

In vitro basophil activation by the mollusc extracts was assessed as described previously.^25^ Briefly, heparinized blood was incubated with the extracts (0.05–5 μg mL^–1^) for 20 min at 37 °C. Basophil activation was assessed by flow cytometry by determining the percentage of viable, high IgE‐expressing, CD63^+^ cells (gating strategy shown in ref. 25). Rabbit anti‐human IgE (Dako) and f‐Met‐Leu‐Phe (fMLP; Sigma, St. Louis, MO) were used as positive controls and OVA (0.05–5 μg mL^−1^) and stimulation buffer alone as negative controls.

### 2.5 SDS‐PAGE, IgE Immunoblot, and IgE Inhibition Immunoblot

Mollusc extract proteins were separated by SDS‐PAGE and stained with Coomassie Brilliant Blue as described previously.^16^ For IgE immunoblotting, mollusc proteins were resolved on a 4–12% Bis–Tris gel (2D‐well, NuPage, Carlsbad, CA) at a concentration of 1 μg protein per millimeter of gel well width and transferred to 0.45 μm nitrocellulose membrane (Thermo Scientific, Rockford, IL). IgE reactivity of separated proteins was determined by sequential probing with patient serum (300 μL per mini‐blotter well, 1:40 dilution), rabbit anti‐human IgE (20 mL per immunoblot, 1:15 000 dilution; Dako) and goat anti‐rabbit IgG‐HRP (20 mL per immunoblot, 1:15 000; Promega) as described previously.^16^

For IgE inhibition immunoblotting, sera (1:30 final dilution) were preincubated for 1 h at room temperature with rPor p 1 (0.4 μg mL^–1^, 2 μg mL^–1^, and 10 μg mL^–1^), OVA (20 μg mL^–1^, negative control), or the same mollusc extract (20 μg mL^–1^) as used for the binding assay (positive control). The preincubated sera were then tested for IgE reactivity with the mollusc extracts by immunoblotting as above. Nonspecific inhibition by mollusc extracts was assessed as described for the inhibition IgE ELISA above.

### 2.6 Identification of IgE‐Reactive Sydney Rock Oyster Proteins by Mass Spectrometry

Bands corresponding to highly IgE‐reactive proteins of raw and cooked SRO extracts with visceral mass were excised from SDS‐PAGE gels for mass spectrometric analysis (Monash Biomedical Proteomics Facility, Monash University, Australia). Briefly, the protein bands were destained, reduced, and alkylated. The gel pieces were washed and dehydrated, followed by trypsin digestion. The gel pieces were then sonicated and analyzed by LC‐MS/MS on a HCT ULTRA ion trap mass spectrometer (Bruker Daltonics, Bremen, Germany) coupled online with an Ultimate 3000 nano HPLC (Dionex Corp., Sunnybrook, CA, USA). Data were exported in Mascot generic file format and searched against the Swiss‐Prot databases using the MASCOT search engine (version 2.1, Matrix Science Inc., London, UK) with all taxonomy selected.

### 2.7 Sequence Analysis of Sydney Rock Oyster Tropomyosin

To determine the amino acid sequence of SRO tropomyosin, cDNA was amplified and sequenced as we described for blue swimmer crab.^16^ Briefly, total RNA was extracted from SRO muscle using TRizol reagent (Life Technologies, Carlsbad, CA, USA) and single‐stranded cDNA was reverse transcribed from the RNA using RT‐PCR (cDNA synthesis kit; Bioline, Sydney, Australia). The tropomyosin coding region was amplified using forward (5ʹ CGC AGA ATT CAT GAC AGC ATC AAG AAG AAG ATG 3ʹ) and reverse (5ʹ CGA ACC TGC AGT TAA TAT CCT GCC AGC TCG G 3ʹ) primers. The primers were designed based on the nucleotide sequence for the closely related Pacific oyster (*Crassostrea gigas*) tropomyosin (GenBank accession number AB444943.1). PCR products were cloned into a sequencing vector, pCR 2.1, using the TOPO TA cloning kit (Life Technologies, Carlsbad, CA, USA) and transformed into TOP10 chemical competent *Escherichia coli*. Positive colonies were confirmed by blue–white screening and colony PCR using gene‐specific oligonucleotide primers for the presence of inserts. Plasmids were purified from overnight culture using an AxyPrep Plasmid Miniprep Kit (Axygen Biosciences, Union City, CA, USA) and sequenced (Macrogen DNA sequencing services, Melbourne, Australia).

### 2.8 Statistical Analysis

The Friedman test in conjunction with Dunn's multiple comparison test was used to compare serum IgE reactivity between mollusc extracts. Differences were defined as statistically significant at *p* < 0.05. The Spearman's rank correlation test was used to assess correlation between IgE ELISA and ImmunoCAP values. Analyses were performed using GraphPad Prism for Windows (GraphPad, San Diego, CA).

## 3 Results

### 3.1 SDS‐PAGE Analysis of Raw and Cooked Mollusc Extracts

**Figure 1:**
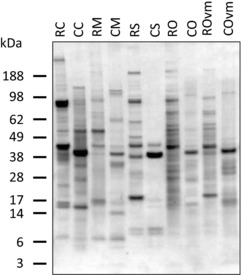
SDS‐PAGE analysis of raw and cooked mollusc extracts. Coomassie Brilliant Blue stained 4–12% reducing SDS‐PAGE gel of raw and cooked mollusc extracts. RC, raw calamari; CC, cooked calamari; RM, raw mussel; CM, cooked mussel; RS, raw scallop; CS, cooked scallop; RO, raw oyster; CO, cooked oyster; ROvm, raw oyster without visceral mass; COvm, cooked oyster without visceral mass.

Analysis of mollusc extracts by SDS‐PAGE revealed a range of proteins from 5 to >188 kDa (**Figure** 1). Protein profiles differed greatly between species, but a prominent protein band was observed at 39–40 kDa for all the cooked extracts, seen at a slightly lower molecular mass (37–39 kDa) and more faintly in the raw extracts, consistent with known tropomyosins (35–39 kDa). There was a pronounced difference between the raw and cooked extract protein profiles with very few protein bands running at the same position in both extracts. Raw extracts from all species contained several higher molecular mass proteins (>140 kDa). These were not detectable after cooking. Conversely, several protein bands appeared in the cooked extracts that were not observed in the raw extracts consistent with heat denaturation of proteins to fragments and production of multimers.

A comparison of oyster extracts prepared with and without the visceral mass revealed a clearer protein profile for the extracts lacking the visceral mass (ROvm and COvm), particularly for the raw extract likely due to removal of endogenous proteinases. However, there was no overall difference in protein profile except for the more prominent band at 20 kDa seen only in the ROvm extract (Figure 1).

### 3.2 IgE ELISA

**Figure 2:**
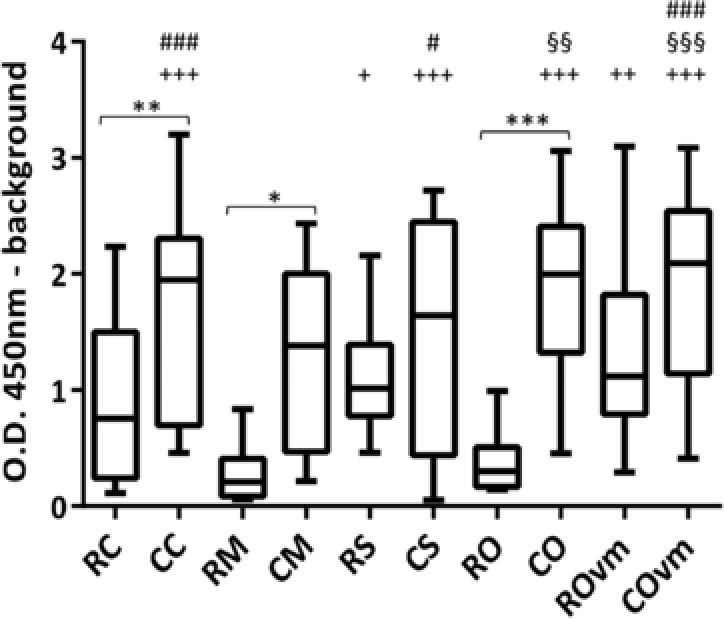
Patient serum IgE reactivity by ELISA. Comparison of serum IgE from allergic patients (*n*  =  13) binding to mollusc extracts showing the median and distribution of O.D. 450 nm. *Comparison between raw and cooked extracts, ^+^comparison to raw mussel extract, ^§^comparison to raw calamari extract, and ^#^comparison to raw oyster extract. ***/+++/§§§/### *p* < 0.001; **/++/§§/## *p* < 0.01 and */+/§/# *p* < 0.05. RC, raw calamari; CC, cooked calamari; RM, raw mussel; CM, cooked mussel; RS, raw scallop; CS, cooked scallop; RO, raw oyster; CO, cooked oyster; ROvm, raw oyster without visceral mass; COvm, cooked oyster without visceral mass.

IgE ELISA showed that all cooked mollusc extracts had higher overall IgE reactivity than their corresponding raw extract, although this difference was not statistically significant for scallop or oyster without visceral mass (**Figure** 2). For individual patients, a positive reaction to raw extract generally corresponded with a positive reaction to the corresponding cooked extract and vice versa. The raw mussel and oyster extracts had lower IgE reactivity than the raw scallop and calamari extracts, but there was no significant difference in IgE reactivity between any of the cooked extracts. Removal of the oyster visceral mass increased IgE binding of the raw extract, but this difference was not seen for the cooked extracts which both showed strong IgE reactivity.

Comparison between patient oyster ImmunoCAP and IgE ELISA values revealed a positive correlation for both raw (*r* ^2^ = 0.7, *p*  =  0.005) and cooked (*r* ^2^ = 0.77, *p*  =  0.0012) oyster extracts. Removal of the visceral mass did not impact this correlation. There was a strong correlation between mussel ImmunoCAP and cooked blue mussel (CM) IgE ELISA values (*r* ^2^ = 0.82, *p*  =  0.002), but interestingly, no correlation for the raw mussel extract. IgE ELISA reactivity for raw or cooked scallop and calamari extracts showed no correlation with respective ImmunoCAP values.

### 3.3 Basophil Activation Test

**Figure 3:**
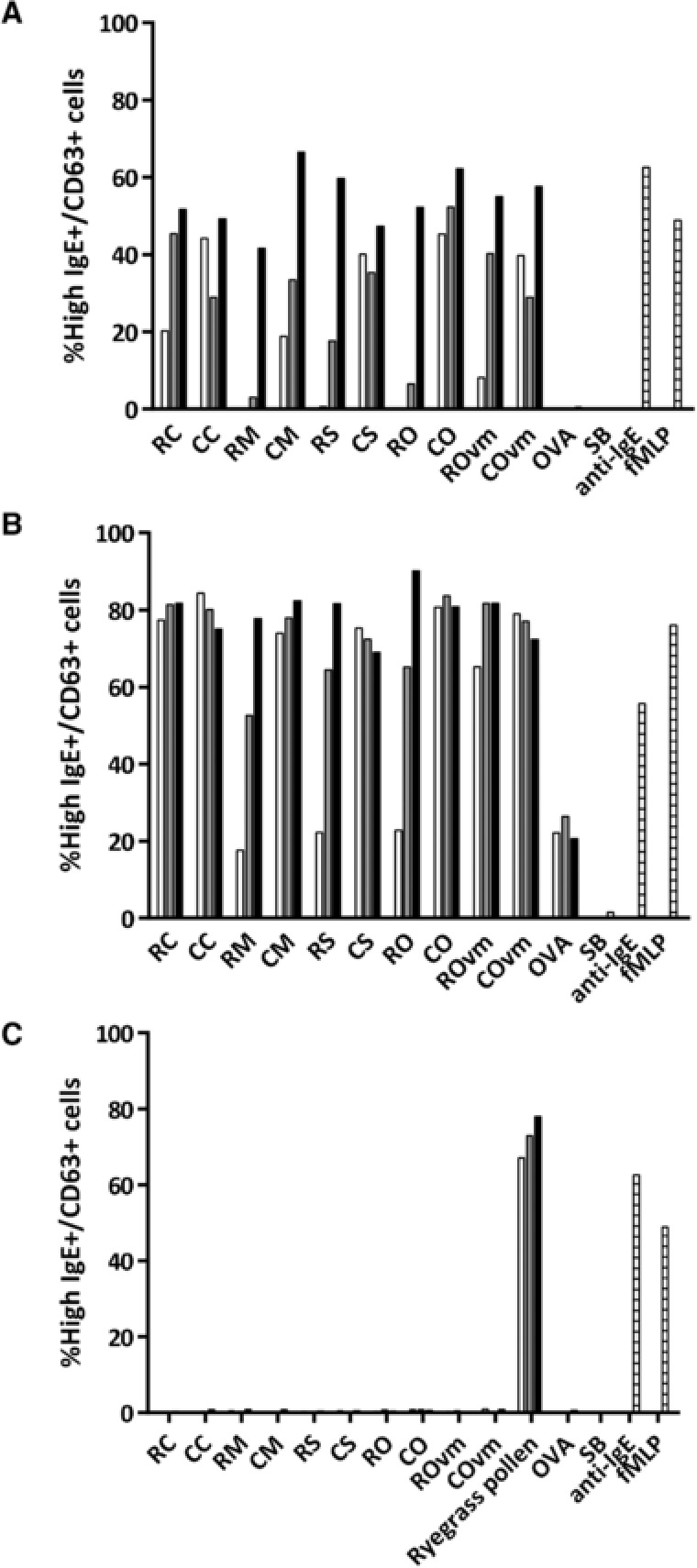
Assessment of functional IgE reactivity to mollusc extracts by basophil activation. Whole blood from patients A) A8, B) A10, and C) a non‐mollusc but ryegrass pollen‐allergic control were stimulated in vitro with mollusc extracts at 0.05 μg mL^–1^ (open bars), 0.5 μg mL^–1^ (grey bars), and 5 μg mL^–1^ (black bars) or controls (striped bars; OVA and SB as negative controls, anti‐IgE and fMLP as positive controls). Basophil activation was assessed by the percentage of live cells expressing high levels of IgE and CD63. RC, raw calamari; CC, cooked calamari; RM, raw mussel; CM, cooked mussel; RS, raw scallop; CS, cooked scallop; RO, raw oyster; CO, cooked oyster; ROvm, raw oyster without visceral mass; COvm, cooked oyster without visceral mass; SB, stimulation buffer.

The biological relevance of mollusc allergen‐specific IgE was assessed by stimulation of basophils in peripheral whole blood from two of the patients (A8 and A10) with the mollusc extracts and flow cytometry (**Figure** 3). Activated basophils were identified by high IgE expression and upregulation of CD63 on the cell surface.^23^ No nonspecific activation or toxicity by the mollusc extracts was observed on testing basophils from a non‐mollusc allergic, atopic subject (Figure 3C). However, there was strong, dose‐dependent activation of basophils from the mollusc‐sensitized subjects, with higher activation for subject A10 compared with A8 and reaching a plateau for some extracts (Figure 3A,B). Again, the cooked extracts induced greater basophil activation than the corresponding raw extracts and removal of the visceral mass from the raw oyster extract increased basophil activation.

### 3.4 IgE Immunoblot

**Figure 4:**
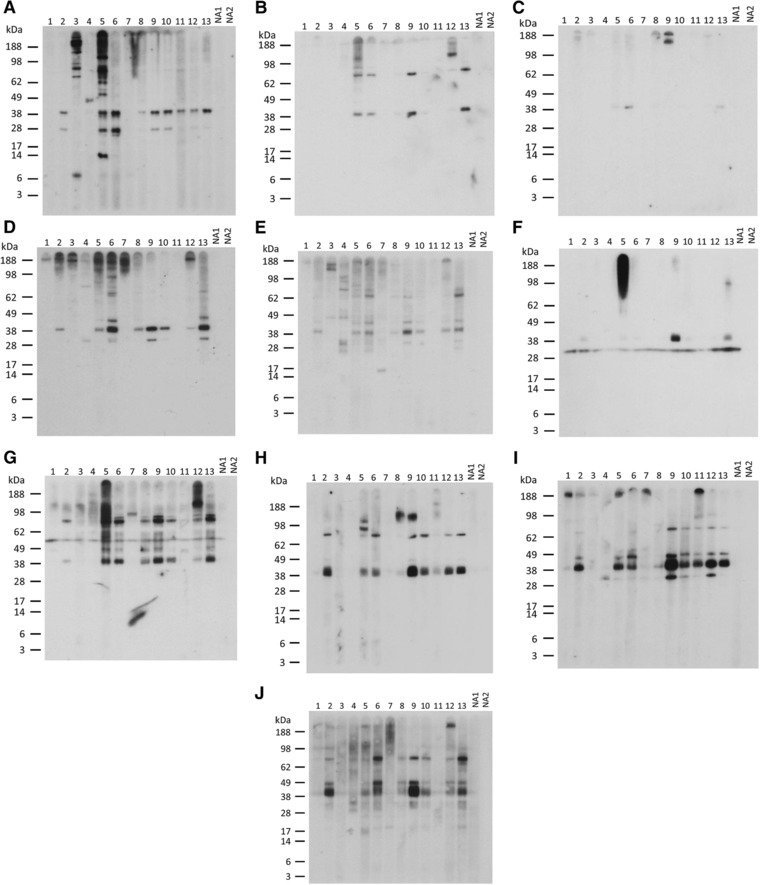
Immunoblot analysis of patient IgE reactivity to mollusc extracts. IgE reactivity to A–E) raw and F–J) cooked mollusc extracts was tested using sera from allergic (1–13) and nonatopic (NA1–2) subjects. A,F) Calamari. B,G) Mussel. C,H) Scallop. D,I) Oyster. E,J) Oyster without visceral mass.

**Figure 5:**
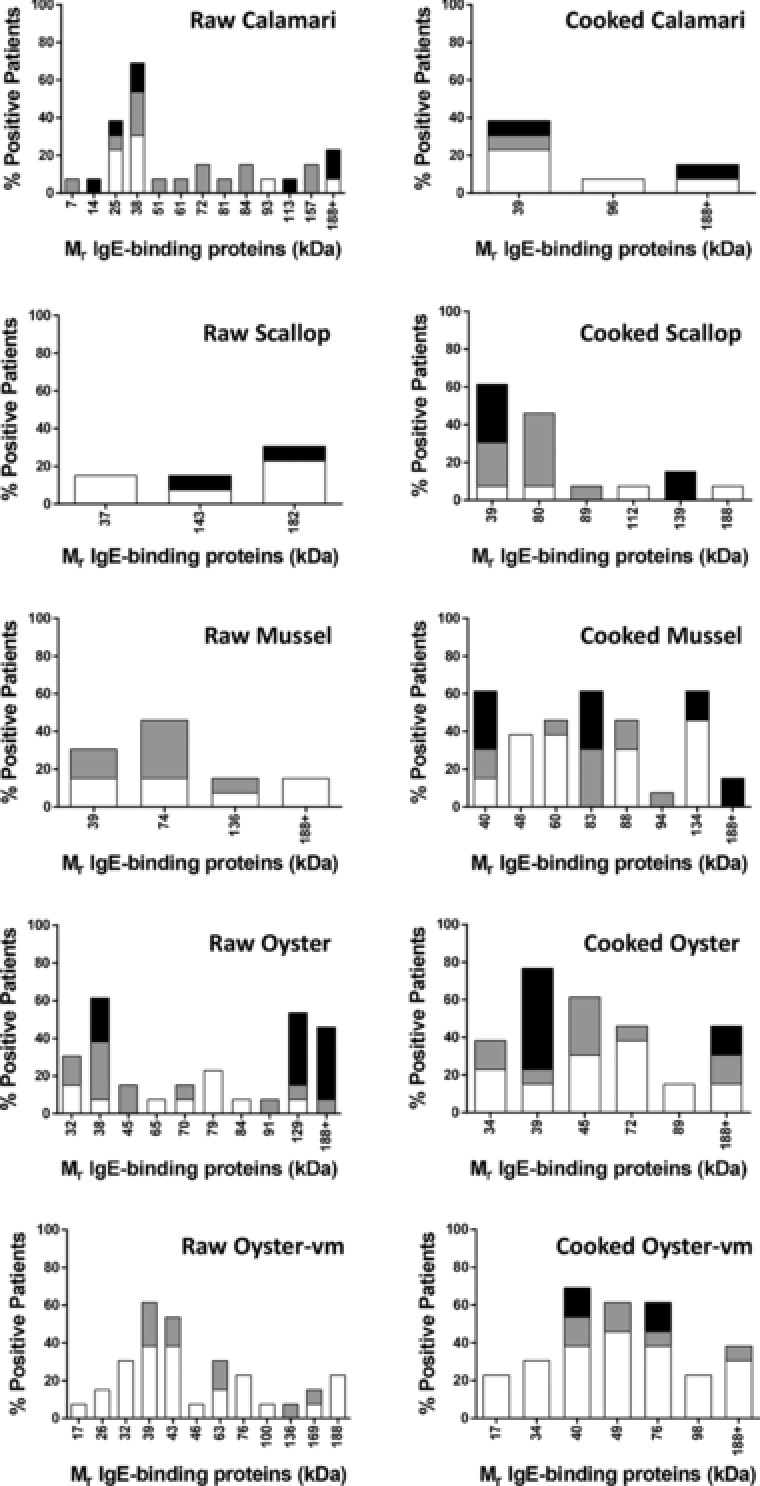
Allergogram analysis of IgE reactivity by immunoblot to mollusc proteins. Percentage of patients with reactivity to IgE‐binding mollusc proteins is shown (*n*  =  13). Intensity of IgE reactivity is graded as weak (white), moderate (gray), or strong (black).

IgE‐reactive proteins within each of the mollusc extracts were examined by immunoblotting (**Figure** 4). The results are summarized in allergograms in **Figure** 5. Overall, there was stronger intensity of IgE binding and a larger number of IgE‐reactive proteins in the cooked extracts compared to the raw extracts, except for calamari where the raw extract was more IgE reactive. The most frequently recognized band was at 37–40 kDa, that is, in the tropomyosin region. Reactivity to this band was >60% for all mollusc species either for the raw, cooked, or both extracts. For scallop, mussel, and oyster extracts, cooking increased the percentage of subjects showing IgE reactivity to this region, with oyster extracts showing the greatest reactivity (CO 69%, COvm 77%). All subjects who showed IgE reactivity to a band in the tropomyosin region in the raw extracts showed IgE reactivity to a slightly lower sized band in the cooked extracts. In the case of calamari, the raw extract showed greater IgE reactivity at this region than the cooked extract (69% vs. 39%). There were several other IgE‐reactive bands recognized at high frequency, especially in the oyster extracts (Figure 5). Proteins from these bands were identified by mass spectrometry as described below.

For the cooked calamari and mussel extracts, the bands seen at 31 kDa and 55 kDa, respectively, for all subjects were shown in control experiments to be due to nonspecific binding of the secondary antibody to the proteins in the absence of patient serum (data not shown).

### 3.5 Inhibition IgE ELISA

**Figure 6:**
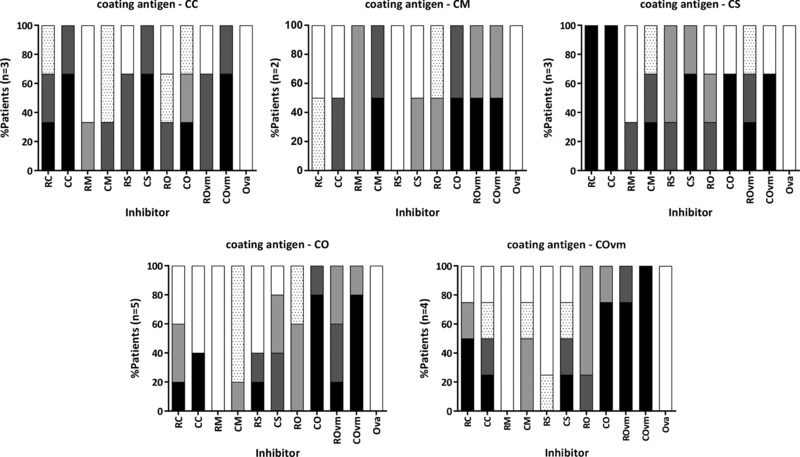
Inhibition IgE ELISA. Inhibition of IgE reactivity to cooked extracts by each mollusc extract is represented by the frequency of patients showing inhibition and the concentration of mollusc inhibitor required to achieve 50% inhibition of IgE reactivity: 0.16–0.8 μg mL^–1^ (black), 0.9–4 μg mL^–1^ (dark gray), 5–20 μg mL^–1^ (light gray), 21–100 μg mL^–1^ (spotted), or >100 μg mL^–1^ (white). Patient sera used for each assay were those showing IgE reactivity to the coating extract by direct ELISA.

Inhibition IgE ELISA was used to assess IgE cross‐reactivity between whole mollusc extracts and the effect of cooking on cross‐reactivity (**Figure** 6). Sera were preincubated with cooked or raw extracts and tested for IgE reactivity with cooked extracts. As expected, inhibition of IgE binding was generally highest when inhibitor and coating extract species were the same. Cooked mollusc extracts consistently showed higher inhibition of IgE reactivity with cooked extracts from other species than raw extracts. Scallop and oyster were strong inhibitors of IgE binding to calamari, and conversely calamari and oyster inhibited IgE binding to scallop strongly. Overall, oyster inhibitors showed the highest cross‐reactivity with other mollusc extracts, with the highest level of inhibition shown to mussel. Confirming specificity of the inhibition ELISA, no inhibition of IgE binding to mollusc extracts was observed in the presence of the negative control inhibitor (OVA; Figure 6) and the mollusc extracts showed negligible nonspecific inhibition of the control non‐shellfish allergic subject serum IgE binding to ryegrass pollen extract (data not shown).

### 3.6 Inhibition IgE Immunoblot

**Figure 7:**
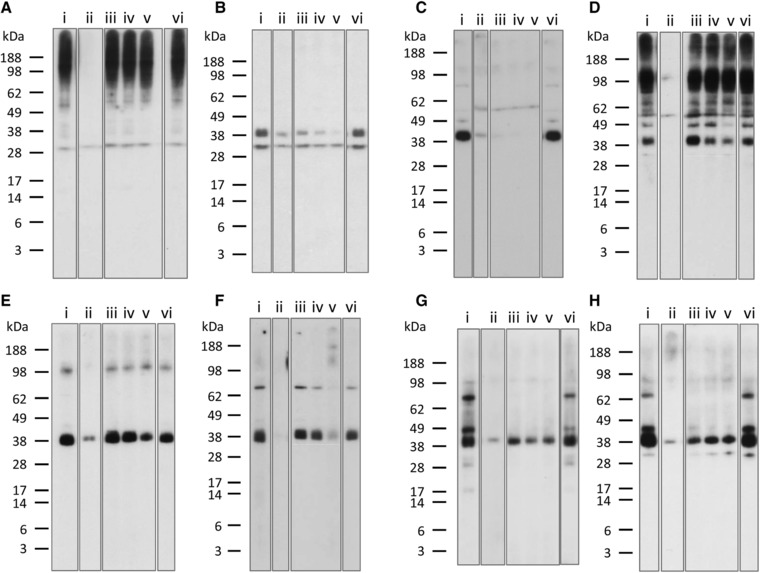
Inhibition IgE immunoblot with rPor p 1. Patient serum was preincubated with rPor p 1 at 0.4 μg mL^−1^ (iii), 2 μg mL^−1^ (iv), and 10 μg mL^−1^, (v) and immunoblotted against: cooked calamari extract A) patient A5, B) patient A9; cooked mussel extract C) patient A9, D) patient A5; cooked scallop extract E) patient A9, F) patient A13; and cooked oyster extract G) patient A12, H) patient A10. No inhibitor (i) and OVA at 20 μg mL^−1^ (vi) were used as negative controls. Same inhibitor as extract was used as a positive control (ii).

Inhibition IgE immunoblotting was used to examine the effect of cooking on mollusc tropomyosin cross‐reactivity with the well‐characterized blue swimmer crab tropomyosin, rPor p 1^16^ (**Figure** 7). Confirming specificity of the inhibition immunoblot assay, for each subject tested there was no inhibition of IgE binding to mollusc extracts by OVA (nonspecific allergen) when compared to the “no inhibitor” control, while whole cooked mollusc extracts were able to completely inhibit IgE reactivity to their respective extracts. The mollusc extracts showed negligible nonspecific inhibition of the control non‐shellfish allergic subject serum IgE binding to ryegrass pollen extract (data not shown).

Two relevant subjects for each cooked mollusc extract, that is, with IgE reactivity to a band in the tropomyosin region, were selected. The crustacean tropomyosin rPor p 1 inhibited IgE reactivity to the “tropomyosin” band for all four mollusc species. In most cases, the inhibition was dose dependent, but for one subject's serum IgE reactivity to mussel extract (Figure 7C) there was complete inhibition at all rPor p 1 concentrations tested. For one serum tested against CC (Figure 7A), there was no reduction in IgE binding to the band at around 30 kDa suggesting that this was not tropomyosin. IgE‐reactive proteins at 30, 48, 67, and 81 kDa in CO, 49 and 81 kDa in CM, and 76 kDa in CS were also inhibited by Por p 1, consistent with cross‐reactive tropomyosin fragments and multimers in the extracts. These findings indicate substantial cross‐reactivity between tropomyosin proteins of mollusc and crustacean shellfish species.

### 3.7 Identification of IgE‐Reactive Sydney Rock Oyster Proteins

To further characterize SRO allergens, we excised IgE‐reactive bands for mass spectrometric identification (**Table** 2). Bands at 34, 39, 45, and 72 kDa were identified as tropomyosin in the cooked oyster extract, consistent with heat‐induced degradation and aggregation. In addition, four proteins were identified from other IgE‐reactive SRO bands: glyceraldehyde‐3‐phosphate dehydrogenase at 38 kDa, fructose‐bisphosphate aldolase and arginine kinase at 45 kDa in the raw but not cooked SRO preparation, and a fragment of myosin heavy chain in the cooked preparation at 89 kDa.

**Table 2:** Proteins identified from IgE‐reactive bands of Sydney rock oyster extracts

Band Molecular mass	Protein name Species Accession no.	Score	Peptide matches	% Coverage
CO1 34 kDa	Tropomyosin *Haliotis rufescens* CAA53028.1	97	2(2)	10
CO2 39 kDa	Tropomyosin *Chlamys nipponensis akazara* O02389.1	579	36(19)	25
CO3 45 kDa	Tropomyosin *Chlamys nipponensis akazara* O02389.1	100	5(2)	19
CO4 72 kDa	Tropomyosin *Chlamys nipponensis akazara* O02389.1	80	2(2)	7
CO5 89 kDa	Myosin heavy chain, striated muscle *Argopecten irradians* P24733.1	191	8(4)	2
RO1 38 kDa	Glyceraldehyde‐3‐phosphate dehydrogenase *Danio rerio* Q5XJ10.2	66	7(2)	5
RO2 45 kDa	Fructose‐bisphosphate aldolase A *Salmo salar* NP_001133181.1	74	1(1)	3
	Arginine kinase *Haliotis madaka* P51544.1	54	1(1)	5

### 3.8 Sequencing and Characterization of Sydney Rock Oyster Tropomyosin

**Figure 8:**
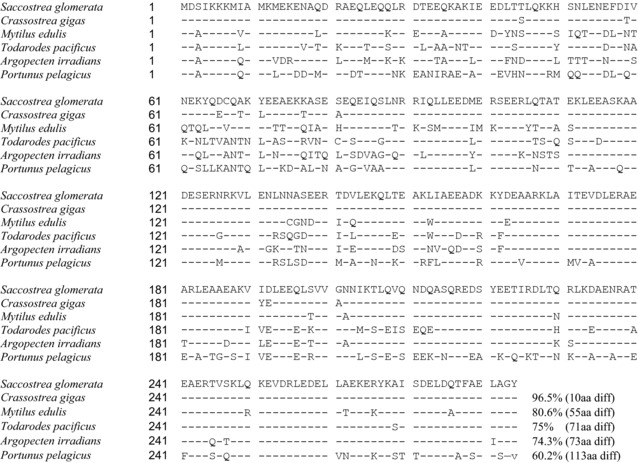
Tropomyosin sequence alignment. Alignment of tropomyosin sequences with *Saccostrea glomerata* (Sydney rock oyster) tropomyosin, Sac g 1 (GenBank accession number MF996471) as reference using NCBI Protein BLAST. Species include the bivalves *Crassostrea gigas* (Pacific oyster; ARX70262), *Mytilus edulis* (blue mussel; Q25457), *Argopecten irradians* (bay scallop; AAX37290), the cephalopod *Todarodes pacificus* (Japanese flying squid; BAE54431) and the crustacean *Portunus pelagicus* (blue swimmer crab; AGE44125). Sequence identity is also shown.

The RNA of the highly IgE‐reactive 38–39 kDa protein from the oyster extracts (as assessed by immunoblot and identified as tropomyosin as above) was cloned and reverse transcribed into cDNA to obtain the complete sequence (**Figure** 8). This allergen has been designated Sac g 1 by the IUIS allergen nomenclature subcommittee (http://www.allergen.org/index.php) based on subsequent further testing of IgE reactivity of the purified protein (data not shown). Alignment of known tropomyosin sequences for different shellfish species shows that SRO tropomyosin has very high sequence identity with tropomyosin from another oyster species (Pacific oyster), and lower identity with two bivalves (blue mussel and bay scallop) and the cephalopod Japanese flying squid (Figure 8). The crustacean tropomyosin Por p 1 had only 60% sequence identity with SRO tropomyosin.

## 4 Discussion

Diagnosis of mollusc allergy is often difficult due to unreliable clinical history as patients may be unaware of the specific trigger of their food‐allergic reaction. Currently, food challenge is the most reliable way to identify food allergy. However, this procedure may be associated with risk of serious adverse reactions in the case of shellfish challenge due to high allergen potency and adult patient comorbidities. For practical reasons, this diagnostic procedure is seldom conducted in adults. A reliable laboratory‐based assay is needed. Hence, we sought to characterize allergens in four commonly consumed Asia‐Pacific mollusc species, assessing frequency of recognition by patient IgE and cross‐reactivity. The effect of heating on IgE reactivity of mollusc extracts was also examined. SRO (*S. glomerata*) tropomyosin, Sac g 1, was sequenced and cross‐reactivity with other mollusc and crustacean tropomyosins assessed.

In order to confirm clinical relevance of IgE reactivity identified by direct IgE binding assays, an effector cell‐based in vitro basophil activation test (BAT) was used. In this assay, native allergen extract was used, presenting conformational as well as linear IgE epitopes. Basophil activation following in vitro allergen challenge as assessed by CD63 expression is a well‐recognized correlate of histamine release and clinical reactivity (e.g., ref.^26^). BAT responses for the two patients tested trended with direct IgE ELISA values and aligned closely with given clinical histories. This test has promising potential for assessing immediate type food allergy in clinical practice.^27^

The correlation of IgE reactivity by ELISA and ImmunoCAP differed between mollusc species. Oyster‐ and mussel‐specific IgE levels by ELISA showed significant correlation with ImmunoCAP values, not seen for scallop and calamari. As both specificity and sensitivity of assays are important for accurate clinical diagnosis, the potency and relevance of allergens used in assay preparations are pivotal. Except for blue mussel, current ImmunoCAP preparations do not include Asia‐Pacific species, likely explaining the limited correlation between our IgE ELISA using locally consumed mollusc species and ImmunoCAP values. Critical to selection of mollusc species for diagnostic assays is knowledge of IgE cross‐reactivity between different mollusc species and between mollusc and crustacean species, as well as the effects of heating on IgE reactivity as we report here. IgE immunoblotting revealed a high frequency of patient IgE reactivity to a heat‐stable 37–40 kDa protein in each of the four mollusc species, consistent with tropomyosin being a major allergen as for other mollusc species.^28^, ^29^, ^30^ We confirmed that this protein was tropomyosin for the highly IgE‐reactive SRO by mass spectrometry and report here, for the first time, the cloning and full sequence analysis of *S. glomerata* tropomyosin, Sac g 1. This revealed strong amino acid sequence identity with another known oyster tropomyosin, but lower identity with tropomyosin from two bivalves and a cephalopod, and even less with the crustacean tropomyosin Por p 1. The level of identity was reflected in the inhibition IgE immunoblotting experiments, where Por p 1 could reduce IgE binding to Sac g 1, but to a lower extent than other mollusc tropomyosins.

In this study, not all subjects showed IgE reactivity to the putative tropomyosin of the four mollusc species. Therefore, tropomyosin‐specific IgE testing alone is insufficient for diagnosis of mollusc allergy. We also observed IgE reactivity to other proteins with molecular masses corresponding to those of known mollusc allergens. These included paramyosin (100 kDa),^31^ hemocyanin (75 kDa),^32^ myosin heavy chain (>208 kDa)^33^ and amylase (60 kDa).^34^ Patient IgE reactivity was also observed to other, previously uncharacterized, proteins. Since the SRO was most IgE reactive in this study, we further characterized potential allergens of this species by excising IgE‐reactive bands for mass spectrometric identification. Tropomyosin was identified at 34, 39, 45, and 72 kDa in the heated extract, consistent with degradation and aggregation on heating. Four other proteins were identified from other bands as putative allergens in SRO. Glyceraldehyde‐3‐phosphate dehydrogenase, fructose‐bisphosphate aldolase, and arginine kinase were identified in the raw but not cooked SRO preparation, consistent with heat‐sensitive allergens as seen for Pacific oyster^15^ and crustacean species,^35^, ^36^ whereas myosin heavy chain was heat stable. Further IgE binding studies are required using purified or recombinant proteins for IUIS allergen designation.

IgE reactivity and allergen sensitization can be affected by food processing.^37^, ^38^ We found that heating increased overall IgE reactivity of mollusc extracts detected by ELISA as we reported previously for crustacean species.^16^, ^39^ That cooked extracts also caused increased basophil activation suggests that increased well coating efficiency was not a major reason for greater reactivity in the ELISA. Likewise, although loss of non‐allergenic components on heating could play a role, the immunoblotting results showed cooked extracts to have a very different profile of IgE‐reactive bands rather than a similar pattern of bands at higher intensity compared with raw extracts. Taken together, our results are consistent with cooking‐induced increased IgE reactivity of certain mollusc proteins.

While many molluscs are cooked before ingestion, thereby potentially presenting heat‐modified allergens to the patient's immune system, this is not usual for oysters. An alternative explanation for the increased IgE reactivity of cooked extracts is that cooking in the presence of endogenous or exogenous sugars results in generation of advanced glycation end products due to the Maillard reaction.^40^, ^41^ Glycated allergens are reported to have enhanced IgE binding.^23^, ^42^ However, there are no clear patterns indicating how different allergens respond to food processing. Our IgE immunoblotting studies showed enhanced reactivity of bands for the cooked mussel, scallop, and oyster extracts, but decreased reactivity for the cooked calamari extract. Different allergen profiles for the species (as shown here) and different endogenous sugars may explain these differing effects of heating. That increased IgE reactivity of calamari on cooking was seen by ELISA and not immunoblotting, might also be due to IgE‐reactive protein aggregation or degradation to small peptides such that they are not observed on the gel but remain in the whole extract for detection by ELISA.

Clinical management of mollusc allergy includes avoidance of the offending species. It is therefore critical to understand the cross‐reactivity between different mollusc species to avoid accidental allergen exposure and potentially severe allergic reactions. Allergen cross‐reactivity between mollusc species and other animals has been suggested both experimentally and clinically.^2^, ^43^, ^44^, ^45^ In this study, the amino acid identity between SRO tropomyosin and other known mollusc tropomyosins could be as low as 74%, suggesting tropomyosin may not be solely responsible for the cross‐reactivity observed.^13^ Our inhibition IgE ELISA studies showed that oyster (a bivalve) had the highest level of overall cross‐reactivity with other mollusc species. Calamari (a cephalopod) and scallop (a bivalve) also demonstrated a high level of cross‐reactivity to each other and with oyster, suggesting taxonomical relativity plays a minor role in mollusc cross‐reactivity. Overall, mussel inhibition was the lowest. These results may reflect differences in abundance of cross‐reactive allergens or epitopes in different mollusc extracts. Our findings suggest that oyster, scallop and calamari‐sensitized patients should be particularly vigilant in avoiding any mollusc species.

Patients with putative mollusc tropomyosin reactivity also demonstrated IgE cross‐reactivity with the major crustacean tropomyosin Por p 1 previously identified by us.^16^ rPor p 1 diminished IgE binding to tropomyosin of cooked calamari, mussel, scallop, and oyster extracts in most subjects. The moderate to high sequence identity between tropomyosins of SRO and other shellfish species suggests IgE epitope similarity as the major determinant of tropomyosin IgE cross‐reactivity. High sequence homology has previously been associated with high affinity and shared IgE binding epitopes of allergens.^46^ Future studies should identify the precise IgE binding regions of mollusc tropomyosins.

Taken together, our novel findings on the characterization of allergenic proteins of mollusc species should inform development of reliable component‐resolved diagnostic assays for mollusc allergy and enable accurate dietary advice.^47^ Diagnostic precision will be enhanced by including the appropriate range of purified allergens from relevant species including those prepared from raw or cooked extracts as appropriate. Coupled with promising advances in delivering hypoallergenic preparations, knowledge of clinically important mollusc allergens provides crucial platform knowledge for the development of much needed safe and efficacious specific immunotherapy for mollusc allergy.^48^, ^49^

## Conflict of Interest

The authors declare no conflict of interest.
